# The Complex Interplay between Lipids, Immune System and Interleukins in Cardio-Metabolic Diseases

**DOI:** 10.3390/ijms19124058

**Published:** 2018-12-14

**Authors:** Stella Bernardi, Annalisa Marcuzzi, Elisa Piscianz, Alberto Tommasini, Bruno Fabris

**Affiliations:** 1 Department of Medical, Surgical and Health Sciences, University of Trieste, Cattinara Teaching Hospital, 34149 Trieste, Italy; stella.bernardi@asuits.sanita.fvg.it (S.B.); b.fabris@fmc.units.it (B.F.); 2 Department of Medical, Surgical and Health Sciences, University of Trieste, 34149 Trieste, Italy; annalisa.marcuzzi@burlo.trieste.it (A.M.); elisa.piscianz@burlo.trieste.it (E.P.); 3 Department of Pediatrics, Institute for Maternal and Child Health-IRCCS “Burlo Garofolo”, 34137 Trieste, Italy

**Keywords:** lipid, innate immune system, interleukin, lipotoxicity, cholesterol, triglyceride, free fatty acids

## Abstract

Lipids and inflammation regulate each other. Early studies on this topic focused on the systemic effects that the acute inflammatory response—and interleukins—had on lipid metabolism. Today, in the era of the obesity epidemic, whose primary complications are cardio-metabolic diseases, attention has moved to the effects that the nutritional environment and lipid derangements have on peripheral tissues, where lipotoxicity leads to organ damage through an imbalance of chronic inflammatory responses. After an overview of the effects that acute inflammation has on the systemic lipid metabolism, this review will describe the lipid-induced immune responses that take place in peripheral tissues and lead to chronic cardio-metabolic diseases. Moreover, the anti-inflammatory effects of lipid lowering drugs, as well as the possibility of using anti-inflammatory agents against cardio-metabolic diseases, will be discussed.

## 1. Introduction

It has been argued that lipid metabolism and immune system responses cannot be separated in real life, they regulate each other, and both are part of a virtuous or vicious cycle/response taking place in the host [1]. This review will describe the interplay between these two systems, whose mediators are lipoproteins (as well as other products such as free fatty acids and lipokines) and interleukins.

**Figure 1:**
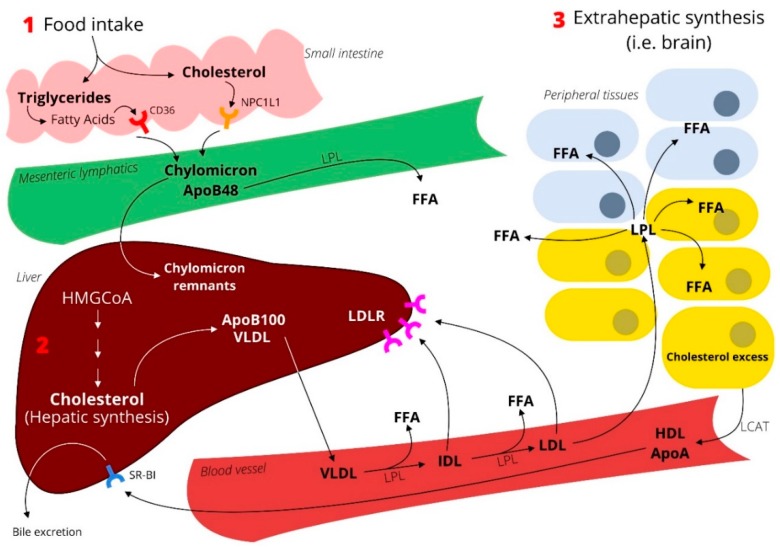
Lipoprotein metabolism. Lipoproteins are classified on the basis of their density as chylomicrons, VLDL, LDL, and HDL. Chylomicrons are low-density lipoproteins that transport dietary lipids from intestinal mucosa to the blood via lymphatic tissue. The associated apolipoproteins include apoA (I, II, IV); apoB48; apoC (I, II, III), and apoE. VLDL transports primarily triglycerides from the liver to the peripheral tissues and its apolipoproteins are apoB100, apoC (I, II; III), and apoE. LDL transports cholesterol esters and its apolipoproteins are apoB100. By contrast, HDL transports cholesterol from the periphery to the liver and it consists of cholesterol esters and its apolipoproteins are apoA (I, II), apoC (I, II, III), and apoE. With respect to the lipoprotein metabolism, after a meal, cholesterol is taken up by the enterocytes via the specific transporter Niemann-Pick C1-Like 1 (NPC1L1). Triglycerides are lipolyzed into free fatty acids (FFA) and taken up either by passive diffusion or by specific transporters such as CD36. Then, cholesterol is esterified by cholesterol acyltransferase and FFA is either re-esterified into triglycerides or released directly into the circulation. Otherwise, cholesterol and triglycerides assemble with apoB48 to form chylomicrons that are released into the circulation. There, they are cleaved by lipoprotein lipase (LPL) into FFA, which is used as an energy source by peripheral tissues. Chylomicron remnants are cleared by liver uptake, through their binding to LDL receptor family members. In parallel, hepatocytes synthesize cholesterol and produce VLDL, which contains triglycerides, cholesterol, and apoB100. VLDL is released into the circulation, where it undergoes lipolysis to release FFA. This becomes LDL and is ultimately cleared away by the hepatic LDL receptor. The reverse cholesterol transport is a process that takes place in the periphery and that is mediated by HDL. Excess cholesterol is transferred to lipid-poor apoAI or to nascent HDL by the specific transporters ATP-binding cassette (ABCA1) and ATP-binding cassette sub-family G member 1 (ABCG1). Next, cholesterol is esterified by lecithin–cholesterol acyltransferase (LCAT). Once HDL is formed, it can directly bind to scavenger receptor class B type 1 (SR-BI) on the liver and transfer cholesterol. Otherwise, cholesteryl esters can be transferred to apoB lipoproteins by cholesteryl ester transfer protein (CEPT), or a small portion of HDL can acquire apoE and bind to LDL receptor.

The term “lipids” refers to lipoproteins, which are circulating macromolecules that transport cholesterol and triglycerides to peripheral tissues. They consist of lipids (triglycerides, cholesterol esters, and free cholesterol) and proteins, called apolipoproteins, which serve as ligands for receptors and as cofactors for enzymes. Specific lipoproteins differ in terms of lipid core content, the proportion of lipids, and type of proteins. Therefore, lipid disorders should be evaluated according to the Friedrickson classification [2], which is based on the pattern of lipoproteins on electrophoresis or ultracentifugation. However, they are most often indicated as hypercholesterolemia (elevated low-density lipoproteins (LDL) cholesterol), hypertriglyceridemia (elevated chilomicrons or very-low density lipoproteins (VLDL) cholesterol), and/or high-density lipoproteins (HDL) cholesterol, which are also the most common lipid disorders. The overview on lipoprotein metabolism is represented in Figure 1.

With respect to interleukins, they are secreted proteins that allow intercellular communication among immune system cells, the immune system and bone marrow cells, and the immune system and peripheral tissues. Since the initial discovery of monocyte and lymphocyte interleukins (IL-1 and IL-2), more than 60 interleukins have been characterized, such that the list goes from IL-1 to IL-39, and includes interferons (IFN) and tumor necrosis factor (TNF) family members. The receptors, functions, and roles of interleukins have been recently reviewed by Akdis [3]. In addition, interleukins are often grouped according to their ability to induce specific T cell differentiation pathways [4]. So far, there are at least seven types of T cell subset differentiation pathways that have been described [4]. These include Th1, Th2, Th17, Th9, Th22, TFH, and Treg responses. Among them, Th1 T cells produce IFN-γ and TNF-α, they activate macrophages and immune responses against intracellular pathogens, while Th2 T cells produce IL-4, IL-5, IL-9, and IL-13, and they drive humoral and IgE mediated immunity [4].

Although lipids and interleukins might seem to be separate systems, they are tightly connected. First, lipids have immune functions against pathogens and modulate immune system responses. Second, interleukins have profound effects on the whole body metabolism, as demonstrated by Hotamisligil in 1993, who showed that TNF neutralization improved insulin sensitivity. These findings provided the scientific evidence that immune mediators are metabolic hormones, a concept also known as immunometabolism [5].

The initial studies that looked at the lipid-interleukin interplay mostly focused on the systemic effects that interleukins and the acute inflammatory response had on systemic lipid metabolism. Consistent with the changes of the global burden of disease [6,7], and given that obesity and its cardio-metabolic complications account for four million deaths globally, scientific attention has progressively moved to the effects that nutritional environment and lipid derangements have on peripheral tissues, where lipotoxicity leads to organ damage through chronic inflammatory responses. In this setting, experimental and clinical studies have demonstrated that IL-1β and IL-18, together with IL-6, IL-8, and TNF-α are key mediators of lipid-induced cardio-metabolic diseases [8,9], while others, such as IL-33 [10], seem to be protective and could be used for therapeutic purposes. 

Having said that, after an overview of the effects that the acute inflammatory response has on systemic lipid metabolism, this review will describe the proinflammatory effects that lipids have on peripheral tissues, leading to atherosclerosis, metabolic syndrome and cardio-metabolic diseases. Moreover, the anti-inflammatory effects of lipid lowering drugs as well as the possibility of using anti-inflammatory agents against cardio-metabolic diseases will be discussed.

## 2. Effects of Acute Inflammation and Interleukins on Lipid Metabolism

The initial studies investigating the relationship between lipids and inflammation focused on the effects that the acute inflammatory response and interleukins had on systemic lipid metabolism. These studies showed that, in humans, acute inflammatory conditions and the rise in the circulating levels of interleukins led to hypertriglyceridemia and hypocholesterolemia [11].

In particular, these studies, which were based on the administration of lipopolysaccharide (LPS) and several interleukins at doses mimicking an acute phase response, showed that hypertrygliceridemia was an early and consistent metabolic alteration taking place in all species during infection/acute inflammation [12,13]. In this setting, the mechanisms underlying VLDL increase included the stimulation of VLDL production, due to both increased hepatic fatty acid synthesis and adipose tissue lipolysis, as well as an impaired VLDL clearance [13]. These effects were obtained by injecting LPS as well as several interleukins, such as TNF [14], whose infusion increased triglyceride levels in both animals [15] and humans [16]. As before, the mechanisms underlying TNF-induced triglyceride increase included not only the inhibition of LPL activity, but also the stimulation of hepatic lipid secretion [17,18,19,20]. Further studies demonstrated that IL-1, IL-2, and IL-6 had similar effects [21], while IL-4 had no effect on hepatic fatty acid synthesis and it actually inhibited IL-1 and IL-6 effects on hepatic lipogenesis [22].

By contrast to triglycerides, these studies showed that cholesterol tended to decrease during an infection/inflammation [12]. This was ascribed to the effects that inflammation and LPS had on cholesterol biosynthesis, as LPS reduced the activity of the enzyme squalene synthase, which is an enzyme necessary for cholesterol biosynthesis. Generally, inhibition of squalene synthase causes a fall in the downstream levels of squalene and cholesterol and an increase in the upstream levels of mevalonate metabolites, which are redirected into other non-sterol pathways [23]. Moreover, infection and/or inflammation reduce apoB secretion, which further decreases serum cholesterol [12].

Interestingly, Feingold et al. put forward that the lipoprotein increase and the lipid changes that were seen during an acute inflammatory response might represent a protective nonspecific immune response—elicited by interleukins—that could decrease the toxicity of harmful biological and chemical agents [11]. Consistent with this view, other studies demonstrated that lipoproteins could bind to endotoxin and that this binding was protective against its deleterious effects [24,25]. In addition, it was also found that lipoproteins could bind to several viruses and reduce their toxic effects [26]. Of note, all these findings are in line with the concept that malnutrition impairs the survival of patients with acute infections [27].

## 3. Effects of Cholesterol on the Innate Immune System and Its Interleukins

### 3.1. Hypercholesterolemia, Inflammation, and Atherosclerosis

Today, moving from infection and malnutrition to overnutrition and cardio-metabolic diseases, the scientific attention has shifted to the effects that hyperlipidemias, such as hypercholesterolemia and hypertriglyceridemia, have on tissue inflammation and chronic cardio-metabolic diseases.

Hypercholesterolemia is associated with the accumulation of LDL in the bloodstream. This condition is very often the result of genetic and environmental factors, like polygenic hypercholesterolemia, but it can also be due to specific genetic disorders, like familial hypercholesterolemia (FH). The first description of FH dates back to 1938, when it was noted that families transmitted hypercholesterolemia as an autosomal dominant trait and this was associated with a dramatic increase in the incidence of cardiovascular disease (CVD) [28]. Then, in 1973, Goldstein and Brown discovered that FH was due to a genetic defect of the LDL receptor (LDLR), resulting in an abnormally low uptake of LDL by the liver [28]. Today, FH is envisioned as a group of related disorders, due to several genetic defects in addition to LDLR mutation [29]. These genetic defects include gain-of-function mutations of *PCSK9* (proprotein convertase subtilisin/kexin type 9), which is a protein that binds to LDLR and promotes its degradation; loss-of-function mutations of *LDLR adaptor protein*, such that LDL fails to be internalized; as well as mutations in the LDLR-binding domain of *apoB100*, or *ABCG5* (ATP-binding cassette sub-family G member 5), and *ABCG8* (ATP-binding cassette sub-family G member 8) [29]. 

According to the “lipid hypothesis” [30], cholesterol levels correlate with the risk of CVD mortality [31], as hypercholesterolemia promotes atherosclerosis development, which is the most common underlying cause of CVD. Rather than being a merely passive accumulation of cholesterol, atherosclerosis is an inflammatory disease [32]. Briefly, cholesterol-containing lipoproteins infiltrate the artery wall, possibly because positive charges on apoB100—as well as apoB48 and apoE—interact with negative charges of proteoglycans of the extracellular matrix with a subsequent lipoprotein retention [33,34]. Then, the subendothelial retention of circulating LDL predisposes the lipids to be oxidized/modified. Once LDL particles are oxidized, they promote endothelial dysfunction, meaning that the endothelium increases its adhesiveness and permeability, and circulating monocytes and T cells are recruited [32]. Although initially macrophage recruitment is meant to protect the vessel by removing modified LDL, when macrophage capacity is overwhelmed, these cells become overloaded with cholesterol ester droplets, they die, and contribute to the formation of a fatty streak with a core full of necrotic debris. At this stage, cells release inflammatory interleukins, chemokines, proteinases, and costimulatory molecules that promote the migration/activation of vascular smooth muscle cells and other immune cells, such that a fatty streak becomes an advanced plaque [32]. All mechanisms, whereby the innate and adaptive immune system promote atherosclerosis development and progression, have been reviewed in detail by Libby and collaborators [35].

**Figure 2:**
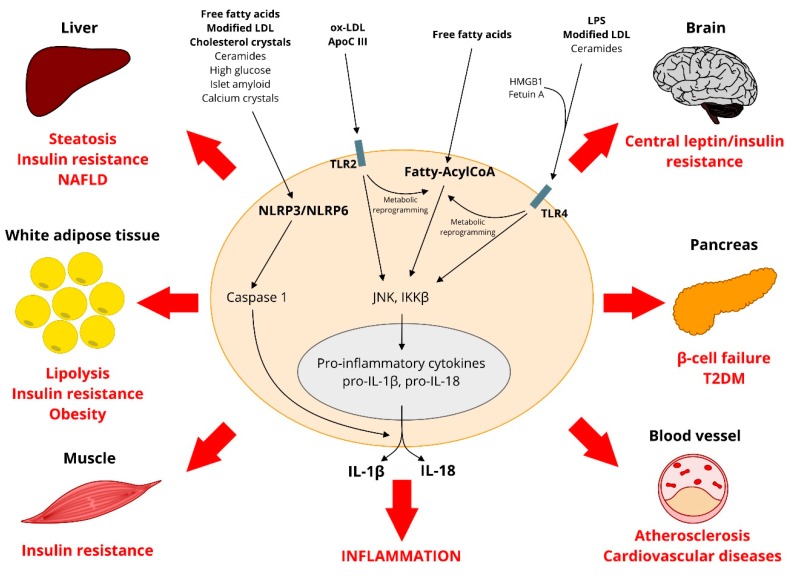
Interaction between lipids and innate immune receptors. Some lipid products, such as oxidized and modified LDL, cholesterol crystals, and ceramides activate innate immune receptors, such as TLR and NLRP3. FFA activate NLRP3, but not TLR. However, TLR-activation is a prerequisite for FFA to induce inflammation. HMBG1 (High Mobility Group Box 1) and fetuin A mediate TLR4 activation. Upon ligand binding, TLR trigger the activation JNK (c-Jun N-terminal kinase) and IKKβ (inhibitor of nuclear factor kappa-B kinase subunit β), leading to the induction of inflammatory gene transcription factors and the expression of proinflammatory cytokines, such as IL-1β and IL-8. NLRP3 activation leads to the expression of proinflammatory cytokines through the assembly of a large multiprotein complex, the inflammasome. The inflammasome consists of the NLRP3 protein, the adapter apoptosis-associated speck-like protein, and pro-caspase-1. The NLRP3-inflammasome catalyzes the cleavage, activation and secretion of IL-1β and IL-18. Inflammation promotes the development of steatosis in the liver, adipose lipolysis, peripheral insulin resistance, leptin resistance in the central nervous system, it impairs insulin secretion in the pancreas, and it promotes the development and progression of atherosclerosis, leading to obesity, non-alcoholic fatty liver disease (NAFLD), type 2 diabetes mellitus (T2DM) and cardiovascular diseases (CVD).

One of the links between cholesterol, the immune system, and atherosclerosis development is the ability that oxidized LDL, modified LDL, and cholesterol crystals have to interact with innate immune receptors and promote tissue damage (Figure 2). Going back to the attempt of macrophages to limit plaque formation, the macrophage scavenger receptors that mediate the internalization of LDL particles include the scavenger receptor A (SR-A), CD36, the macrophage receptor with collagenous structure (MARCO), and the lectin-like ox-LDL receptor-1 (LOX1). Interestingly, studies on hypercholesterolemic mice have provided contradictory results on the exact role of scavenger receptors in atherosclerosis, possibly because they participate in the process of cholesterol efflux from tissues [36]. By contrast, experimental studies highlight a major proatherosclerotic role for multiple innate immune receptors that have been implicated in the recognition of metabolic stimuli/stressors, such as LDL particles, and the initiation of inflammatory responses in peripheral tissues through IL-1β and IL-18 secretion [9] (Figure 2). The typical example is represented by toll-like receptors (TLR) [37], which are innate immune system effectors that are usually required for a host defense against pathogens. The best known is TLR4, which binds to LPS on the bacterial cell wall and triggers the synthesis of many proinflammatory proteins [38,39]. Both the endothelium and monocyte-derived macrophages express a broad range of TLR [40] that can interact with different types of LDL particles. First, oxidized LDL can bind to TLR2 on the vasculature and induce vascular inflammation [41]. Interestingly, in atherosclerosis-susceptible *LDLR*-knockout mice, complete deficiency of *TLR2* led to a reduction in atherosclerosis. However, loss of *TLR2* expression from bone marrow-derived cells had no effect on disease progression, indicating that only TLR2 on vasculature is proatherogenic [42]. Second, it has been shown that modified LDL can bind to TLR4 and promote atherosclerosis [43]. Interestingly, even though *TLR4* deficiency reduced atherosclerosis extent, the lack of *MyD88*, an adaptor protein in the TLR signaling cascade, further reduced it [44,45], possibly because it participates in the signal-transduction pathway of the receptors for IL-1β and IL-18 [46,47]. Third, cholesterol crystals can bind to the intracellular *NLRP3* (nucleotide-binding oligomerization domain-like receptor pyrin domain-containing 3), which is another type of immune receptor. This interaction leads to the secretion of IL-1β and IL-18 [48], which have been associated with atherosclerosis severity [49]. Interestingly, deletion of *NLRP3* markedly reduced the development of aortic lesions in *LDLR*-knockout mice [50]. Based on this evidence, it is current scientific opinion that IL-1β and other IL-1 family cytokines are key vascular and systemic inflammatory mediators that significantly contribute to atherogenesis [8].

It has been argued that the systemic inflammatory response can promote atherosclerosis development [32]. Consistent with this concept, it has been shown that IL-1β and TNF-α promote LDL binding to various cell types in vitro [51]. Also C-reactive protein (CRP), which is an acute phase reactant and a useful biomarker of CVD, might contribute to atherosclerosis development [52]. For instance, it has been demonstrated that CRP binds to oxidized LDL and oxidized phospholipids, and it promotes their uptake by macrophages [53]. Moreover, recent studies support a causal role for IL-6 signaling pathway in atherosclerosis [54], which is supported by the evidence that modulation of the IL-6 pathway associates with reduced cardiovascular events [54]. Moreover, hypercholesterolemia appears to be associated with systemic inflammation, as assessed by CRP measurement [55]. For example, patients with homozygous FH exhibit higher levels of CRP, IL-1β, IL-2R, IL-6, IL-8, IL-10, and TNF-α [56]. Furthermore, these proinflammatory changes in patients with FH contribute to endothelial dysfunction and atherosclerosis development independent of cholesterol levels [57]. Interestingly, hypercholesterolemia seems also associated with the expansion of circulating monocytes and neutrophils [58], increased migratory capacity [59], and several works indicate that hypercholesterolemia promotes the proliferation and mobilization of hematopoietic stem cells, as well as extramedullary hematopoiesis [60].

### 3.2. Reduction of Inflammation, LDL, or Both to Protect from Cardiovascular Diseases

According to the “lipid hypothesis” [30], which suggests that there is a linear relationship between cholesterol and the risk of CVD morbidity [31], meaning that cholesterol reduction lowers CVD [30], much scientific effort has been put into effective ways to lower blood cholesterol. Statins, which are 3-Hydroxy-3-methylglutarylcoenzyme A (HMG-CoA) reductase inhibitors, are the first-line drugs for patients with hypercholesterolemia. They inhibit HMGCoA reductase, which is the rate-limiting enzyme for cholesterol synthesis, whereby they reduce lipoprotein release from the liver. In addition, they upregulate *LDLR* expression and enhance LDL clearance. Of note, other drugs that decrease lipoprotein production by the liver include the apoB antisense oligonucleotide, mipomersen, and the microsomal triglyceride transfer inhibitor, lomitapide, which are restricted to patients with homozygous FH [61,62]. Alternative treatments include the cholesterol absorption inhibitor ezetimibe and the inhibition of PCSK9 [63].

In 1994, the Scandinavian Simvastatin Survival Study (4S) demonstrated that giving simvastatin to patients with pre-existing coronary artery disease and high LDL significantly decreased cardiovascular and total mortality rates, providing compelling evidence of the benefit of cholesterol reduction [64]. After the 4S study, other interventional studies have clearly demonstrated that statins safely reduce CVD morbidity and mortality in high-risk patients in primary and secondary prevention, irrespective of initial LDL [65]. Today, based on the evidence that cholesterol levels correlate with the risk of coronary heart disease mortality [31], target levels are defined as those associated with minimal cardiovascular risk rather than population averages. By contrast, controversies exist regarding the association between triglycerides and CVD, as triglyceride levels are not independently associated with coronary mortality [66]. Nevertheless, lowering triglyceride levels <200 mg/dL ameliorates atherosclerotic disease in patients with well-controlled LDL [67]. Thus, triglycerides should be less than 150 mg/dL [68,69]. Having said that, with respect to cholesterol, guidelines recommend that total cholesterol should be less than 200 mg/dL in the general population. In addition, after stratifying the population according to their cardiac risk, guidelines recommend that LDL should be less than 130 mg/dL in low risk patients, less than 115 mg/dL in moderate risk patients, less than 100 mg/dL in high risk patients, and less than 70 mg/dL in very high risk patients. 

In 2008, the JUPITER trial demonstrated that in healthy people without lipid abnormalities but with elevated CRP levels, rosuvastatin significantly reduced inflammation and the incidence of cardiovascular events [70]. These results raised the question as to whether the clinical benefits of statin treatment were due to LDL, inflammation, or the reduction of both. As matter of fact, statins have direct anti-inflammatory effects, which have been ascribed to the interaction with several cellular targets, including small G proteins, such as Ras, Rac, and Rho [71], as well as the promotion of the expression of antiatherosclerotic molecules [72]. In addition, the JUPITER trial speculated that lowering inflammation could lower CVD, independent of cholesterol levels. To test this hypothesis, two interventional studies have been recently designed [73]. The first one, called Canakinumab Anti-inflammatory Thrombosis Outcomes Study (CANTOS) demonstrated that canakinumab, a monoclonal antibody against IL-1β, significantly reduced CVD in the absence of lipid lowering effects [74], possibly by modulating the IL-6 signaling pathway [54]. As a proof-of-concept study supporting the inflammatory hypothesis of atherosclerosis, the CANTOS study has been a success. Nevertheless, given that the group of patients treated with canakinumab had a higher—although not statistically significant—incidence of fatal infections, other therapeutic agents/strategies and more realistic choices are needed [65]. Meanwhile, the second interventional study, the Cardiovascular Inflammation Reduction Trial (CIRT), which evaluates whether methotrexate will reduce CVD, is still ongoing [73].

The inhibition of PCKS9 is an alternative therapeutic option for lowering cholesterol in patients where statin therapy fails. Two recent trials, the FOURIER and the ODYSSEY OUTCOMES trials, have demonstrated that PCKS9 inhibition significantly reduced the risk of major adverse cardiovascular events in very high risk patients whose LDL remained elevated despite statin therapy at the maximum tolerated dose [75,76]. PCSK9 is a protein of 692 amino acids belonging to the proprotein convertase family, whose major function is LDLR degradation by binding to it both intracellularly and at the cell surface. Currently, there are two monoclonal antibodies to PCSK (alirocumab and evolocumab) that are available in clinical practice. They inhibit the interaction between PCSK9 and the LDLR, leading to an increase in the number of LDLR and ultimately enhancing LDL uptake by the liver [63]. Alternative ways to inhibit PCSK9 include small interfering RNA (siRNA), vaccines, antisense oligonucleotides, and small molecule inhibitors [63]. Interestingly, the advantages of PCSK9 inhibition go beyond LDL level reduction. For example, PCKS9 inhibition reduces lipoprotein(a) (Lp(a)) [77,78], which is similar to LDL but more atherogenic. In addition, recent studies suggest that PCSK9 inhibition might have anti-inflammatory effects, independent of LDL cholesterol levels [79]. For instance, Lan and colleagues showed that PCSK9 affects multiple pathways beyond cholesterol metabolism, such as the cell cycle, the xenobiotic metabolism, and the inflammation and stress response pathways [80]. Interestingly, when *PCSK9*-knockout mice were injected with LPS they exhibited a blunted inflammatory response, as the levels of TNF-α and IL-6, as well as other mediators, were significantly lower as compared to wild-type mice [81]. Although *PCSK9* is mainly expressed by the liver, it can be found in extrahepatic tissues too, like in the vasculature, such as in endothelial cells, vascular smooth muscle cells, and monocytes/macropahges [79]. At a tissue level, *PCSK9* deficiency significantly reduced LOX-1 (Lectin-like oxidized LDL receptor-1) expression [82]. Moreover, consistent with a proatherosclerotic role for PCKS9 independent of lipid levels, *PCKS9* overexpression accelerated atherosclerosis in *apoE*-knockout mice without significantly affecting plasma lipid levels [83], while gene inactivation significantly reduced it [84].

### 3.3. The Lipid Paradox

An observation that challenges the lipid-inflammation paradigm is the fact that patients with some inflammatory diseases, such as rheumatoid arthritis (RA), may exhibit a fall in LDL cholesterol, but they still have an increased CVD risk [85]. An explanation for this lipid paradox is that chronic low-grade inflammation has effects on the cardiovascular system that are independent of lipid levels, consistent with the JUPITER and CANTOS studies. Another explanation is that inflammation promotes some changes of the composition/quality of lipoproteins subfractions, ultimately promoting atherosclerosis development. For example, TNF-α and IL-6 promote the oxidation and the uptake of oxidized LDL by macrophages, favoring their transformation into foam cells [86]. In addition, patients with RA exhibit higher levels of small dense LDL particles that cross the endothelium more easily and are more prone to oxidative changes [87,88], as well as higher levels of Lp(a) [89]. Conversely, serum levels of protective small HDL are diminished [87]. Consistent with the lipid paradox, patients with RA who were treated with anti-TNF-α therapy exhibited a 10% increase in total cholesterol and a 7% increase in HDL within 6 months of TNF-α blockade [90]. However, the same TNF-α blockade has been associated with substantial morbidity and mortality benefit [91,92]. To reconcile these findings, it has been argued that TNF-α inhibition changes the quality of lipoproteins, as it ameliorates the antioxidative capacity of HDL regardless of their concentrations [93], and it leads to a dose-dependent reduction in serum Lp(a) [94].

### 3.4. A Defective Cholesterol Biosynthesis Triggers Inflammation

**Figure 3:**
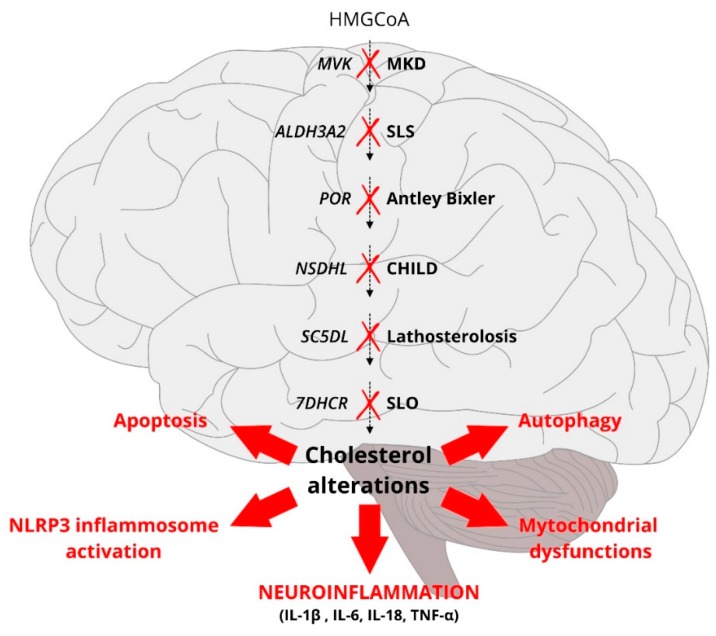
Cholesterol biosynthesis deficiency syndromes. Schematic representation of the cholesterol pathway and some cholesterol–deficiency syndromes in response to different enzyme defects along the metabolic pathway (indicated with red crosses). Despite different specific causes, all these syndromes share the involvement of the central nervous system, where cholesterol reduction causes NLRP3-inflammosome activation, apoptosis, mitochondrial dysfunctions, autophagy and neuroinflammation with interleukin secretion (IL-1β, IL-18, IL-6 and TNF-α). HMG-CoA: 3-Hydroxy-3-MethylGlutaryl Co-enzyme; *MVK*: mevalotate kinase gene; MKD: Mevalonate Kinase Deficiency; *ALDH3A2*: Aldehyde Dehydrogenase 3 Family Member A2 gene; SLS: Sjogren-Larsson syndrome; *POR*: Cytochrome P450 Oxidoreductase gene; Antley-Bixler syndrome-like phenotype with disordered steroidogenesis; *NSDHL*: NAD(P) Dependent Steroid Dehydrogenase-Like gene; CHILD: congenital hemidysplasia with ichthyosiform erythroderma and limb defects; *SC5DL*: Sterol-C5-Desaturase gene; Lathosterolosis; *7DHCR*: 7-Dehydrocholesterol Reductase gene; SLO: Smith-Lemli-Opitz) syndrome; IL-1β: Interleukin 1 beta; IL-6: Interleukin 6; IL-18: Interleukin 18; TNF-α: Tumor necrosis factor alpha.

Another observation that challenges the lipid-inflammation paradigm is that patients with defective cholesterol biosynthesis suffer from central nervous system disorders due to neuroinflammation. As a foreword to these disorders, it must be said that hypocholesterolemia is generally secondary to systemic diseases and one of its most common causes is sepsis (due to the mechanisms described in the first part of the review). Several works have reported that critically ill patients and/or patients with sepsis, exhibit low cholesterol and LDL values, which are inversely related to circulating interleukins, such as IL-2, IL-6 and IL-10 [95,96]. Less frequently, hypocholesterolemia is due to genetic disorders, and it is a rare occurrence, with the exception of familial hypobetalipoproteinemia. Familial hypobetalipoproteinemia is defined as apoB and LDL levels below the 5th percentile and seems to be due to several defects, including loss-of-function mutations of *PCSK9* and/or mutations leading to truncation of apoB. Other primary forms include abetalipoproteinemia, characterized by a virtual absence of apoB-containing lipoproteins, the Anderson disease, or chylomicron retention syndrome, and combined familial hypolipidemia, which is due to a mutation of the gene for the angiopoietin-like 3 (*ANGPTL3*) protein. In addition to these forms of “systemic” hypocholesterolemia, there can be “tissue” hypocholesterolemias, such as in patients with inherited defects in cholesterol biosynthesis [97], where circulating cholesterol is normal or slightly low but brain cholesterol can be virtually absent. This is due to the fact that all the cholesterol contained in the brain is the result of an in situ synthesis because the blood-brain barrier does not allow the passage of circulating lipoproteins [98,99,100]. Therefore, when there is a defect in cholesterol biosynthesis and cholesterol synthesis is impaired, intermediate metabolites accumulate in the central nervous system and cause apoptosis, autophagy, and NLRP3-inflammasome activation [97,100,101,102] (Figure 3), leading to neuroinflammation [98,99,103]. The damage to the central nervous system presents with psychomotor retardation, developmental delay, structural brain malformations, multiple congenital anomalies, microcephaly, autism and other behavioral disorders [97]. 

## 4. Effects of Triglycerides on the Innate Immune System and Its Interleukins

### 4.1. Hypertriglyceridemia: A Case for a Proinflammatory Condition

Not only hypercholesterolemia, but also primary isolated hypertriglyceridemia has been associated with systemic inflammation. Hypertriglyceridemia results from the accumulation of triglyceride-rich lipoproteins (e.g., VLDL, VLDL remnants, and chylomicrons) in the bloodstream. This condition is very often the result of genetic and environmental factors but it can result also from specific genetic disorders, like hypertriglyceridemia, familial combined hyperlipidemia, familial dysbetalipoproteinemia, and familial chylomicronemia [104]. Nevertheless, 33% of the adult population is estimated to have serum triglycerides higher than 150 mg/dL, and this percentage increases in the subjects aged 60 years or older [105]. 

In particular, several reports have shown that patients with isolated hypertriglyceridemia had increased CRP concentration [106,107]. This was associated with high levels of IL-6 [106,107], which seems to be the single most important factor controlling the hepatic acute-phase response [108]. Other works have reported an association between hypertriglyceridemia and IL-8, MCP-1, and TNF-α [106,109]. This association has been known for a long time and it can be ascribed to the fact that triglyceride-rich lipoproteins seem to be proinflammatory [110]. In particular, one of the events whereby triglycerides might induce tissue inflammation is the lipolysis that takes place in the postprandial state, when triglycerides release FFA. It has been shown that there is a 10-fold increase in FFA concentration when triglycerides are incubated with lipoprotein lipase (LPL) [111], and that FFA induce endothelial proinflammatory changes, as assessed by TNF-α and ICAM production, as well as reactive oxygen species formation in cultured endothelial cells [112]. Consistent with the effects that postprandial lipoproteins might have on the vasculature [113], it has been recently shown that also fasting triglycerides, which are used as a predictor of the postprandial levels, are independent predictors of atherosclerosis and cardiovascular events [114,115]. 

### 4.2. Free Fatty Acids, Tissue Inflammation, Metabolic Changes

In 1993, the landmark study by Hotamisligil and Spiegelman demonstrated that TNF-α neutralization ameliorated the peripheral response to insulin, linking inflammation to metabolism [116]. Saturated FFA (such as palmitic acid) have emerged as one of the connectors between hypertriglyceridemia, chronic tissue inflammation and metabolic disturbances. FFA can trigger inflammation on several peripheral tissues. For instance, palmitate increased myocyte [117] and adipocyte [118] expression of IL-6, TNF-α, and we have recently reported that palmitate increased CXCL8 and TNF-α on hepatocytes [119]. Most importantly, experimental evidence shows that it is through tissue inflammation that FFA promote insulin resistance in peripheral tissues, such as skeletal muscle [120] and adipose tissue [121]. For example, the inhibition of skeletal muscle nuclear factor kappa-B (NF-κB) activation prevented FFA-induced insulin resistance [120]. Likewise, the knockdown of stress/inflammatory kinases c-Jun N-terminal kinase (JNK) and inhibitor of NF-κB kinase subunit β (IKKβ) prevented FFA-induced insulin resistance in adipocytes [121]. Interestingly, FFA can trigger an inflammation also on other tissues, such as endothelial cells [122], vascular smooth muscle cells [123] and/or circulating monocytes [124].

Similar to what concerns the uptake of cholesterol in the vasculature, studies on the specific FFA receptors, which include CD36 and fatty acid transport protein (FATP1), have provided contradictory results on their exact role in tissue inflammation and metabolic changes. Both their upregulation and deficiency may cause tissue damage by disrupting homeostasis [125,126,127]. By contrast, several innate immune receptors have been implicated in the response to FFA, and in the initiation of proinflammatory changes in peripheral metabolic tissues [9] (Figure 2). Again, these innate immune receptors include TLR as well as nucleotide-binding oligomerization domain receptors (NOD1/2) and NLRP3. Among them, TLR are the best characterized family of receptors. They regulate the innate immune system response by activating proinflammatory signaling pathways in response to microbial pathogens [37]. The hypothesis that FFA could activate TLR4 relied on the observation that saturated FFA was necessary for LPS-induced inflammation [128,129]. Further studies have led to the current view that different pathogens—as well as danger—associated molecular patterns (PAMPs or DAMPs) can induce inflammatory reactions on macrophages and other immune cells, by binding to these pattern recognition receptors (such as TLR, NOD and NLRP3), with subsequent release of IL-1β and IL-18 [9]. Interestingly, it has been clarified that palmitate is not an agonist for TLR4 and it does not activate TLR signaling. However, TLR activation is crucial for FFA-induced inflammation, because TLR priming leads to a metabolic reprogramming that promotes palmitate-induced inflammatory changes [130]. Interestingly, unsaturated FFA inhibit saturated FFA-induced proinflammtory effects [131]. Furthermore, the binding of NLRP3 to FFA or to unsaturated/omega-3 fatty acids has opposite effects, which are proinflammatory in the first and anti-inflammatory in the second case [132]. Anyhow, other lipoprotein components/products can bind to innate immune receptors and triggers proinflammatory responses, such as apoCIII that can interact with TLR2 and activate circulating monocytes [133].

Given that TLR4 is expressed ubiquitously, TLR4 has emerged as an important mediator of metabolic inflammation [37]. For example, Shi et al. have been one of the first to demonstrate that FFA activated TLR signaling on adipocytes, with subsequent release of IL-6 and TNF-α, and that mice lacking *TLR4* displayed lower expression of proinflammatory molecules and greater insulin sensitivity, when fed with a high-fat diet [134]. Further works have consistently shown that TLR4 deficiency reduced inflammation, as well as insulin resistance, and hepatic steatosis in response to a high-fat diet [135,136,137,138,139]. Interestingly, it has been proposed that *TLR4* could directly influence adipose tissue macrophage polarization, which could be a mechanism promoting adipose tissue changes in obese patients [124,139]. Emerging evidence has revealed that obesity and diabetes increase TLR expression, promoting tissue inflammation [140]. For example, Jialal et al. have found that TLR tissue expression increases in diabetic patients [141,142], possibly induced by hyperglycemia [143], and that FFA amplifie monocyte inflammation in high glucose conditions [144], which could justify hyperglycemia-induced inflammation. Consistent with this data, human and animal studies show that obesity and insulin resistance are associated with increased NLRP3 expression in the adipose tissue [132]. 

### 4.3. Hypertriglyceridemia is a Feature of the Metabolic Syndrome

Having said that, although hypertriglyceridemia can be an isolated occurrence, it is very often secondary to obesity and diabetes mellitus. With respect to obesity, the mechanisms leading to hypertriglyceridemia in patients with diet-induced obesity include increased secretion and severely impaired clearance of triglyceride-rich particles [145]. It has been demonstrated that in obese subjects without overt hyperlipidaemia, chylomicron catabolism is impaired [146], and these patients display a combination of overproduction of VLDL-apoB particles and decreased catabolism of apoB containing particles in patients with visceral obesity [147]. With respect to diabetes, given that insulin stimulates LPL activity and inhibits hormone-sensitive lipase in the adipose tissue, circulating chylomicrons increase as well as the amount of FFA that are released by the adipose tissue, which facilitate hepatic VLDL production [148]. Moreover, obesity and diabetes are often associated with chronic low-grade inflammation in peripheral tissues, which impairs lipid metabolism, and activates immune responses, in the setting of the so-called metabolic syndrome [149]. 

The metabolic syndrome is a condition where obesity is associated with a chronic low-grade inflammation, which leads to impaired fasting glucose or type 2 diabetes, hypertryglyceridemia, low HDL cholesterol, hypertension, [150], as well as non-alcoholic fatty liver disease (NAFLD) and CVD. The description of NAFLD pathogenesis is beyond the scope of this review and can be found in a recent review by [151]. Today, the metabolic syndrome is one of the most common metabolic disturbances affecting 20–25% of the world’s population [150]. With respect to metabolic syndrome development, it is current scientific opinion that in obesity, the capacity of the adipose tissue to expand is overwhelmed by overnutrition. Adipocytes increase both in size and number, and this leads to local hypoxia, adipocyte death, and local inflammation [152] with drastic changes in the resident immune cell profile and function. Specifically, obese mice and humans accumulate macrophages in their adipose tissue, proportionally with the increase of their body mass index [153,154]. Moreover, macrophages shift from the anti-inflammatory phenotype of the lean state to the pro-inflammatory phenotype of obesity. In the lean state, resident adipose tissue macrophages (also called M2 macrophages) together with Th2 T cells, Treg cells, and eosinophils produce anti-inflammatory cytokines such as IL-4, IL-10, and IL-13 [155], which maintain insulin sensitivity. As compared to the lean state, obesity promotes macrophage recruitment, as well as their shift from M2 to the harmful M1 macrophage phenotype [156]. This shift leads to a relative decrease of anti-inflammatory IL-4, IL-10, and IL-13 and to an increased production of TNF-α, IL-1β, IL-6 in the adipose tissue, which altogether promote tissue and systemic inflammation and insulin resistance [157]. The rationale and the results of targeting inflammation in the treatment of diabetes are discussed by Donath in Reference [158]. Moreover, proinflammatory cytokines such as TNF-α regulate lipid metabolism in adipocytes via increasing lipolysis and FFA release [159].

Not surprisingly, a great number of experimental and clinical studies have shown that metabolic syndrome is directly associated with circulating IL-6, TNF-α, and CRP [160,161,162], and that it is inversely correlated with IL-10 levels [163]. In addition, it has been demonstrated that circulating IL-6 is associated with body fat and insulin resistance [164,165]. Consistent with these findings, we reported that experimental models, such as the high-fat diet-fed mouse, as well as patients with metabolic syndrome displayed a significant increase of circulating IL-6 and CRP [119,122,166,167], and that the high-fat diet milieu was directly implicated in IL-6 changes as shown in vitro experiments [122]. Interestingly, although elevated levels of proinflammatory mediators are thought to be the consequence rather than the cause of obesity, it has been shown that also the reverse can be true, as subjects with elevated concentrations of inflammatory markers were more prone to gain weight during the follow-up of patients recruited for the MONICA study [168]. 

The concept that cardio-metabolic diseases are due to an imbalance of chronic inflammatory responses relies on the observation that several Th2 cytokines, such as IL-4, IL-5, and IL-13, which usually decrease in obesity, are those that generally improve glucose tolerance and insulin sensitivity [169]. As a result, their administration should reverse the metabolic abnormalities induced by overnutrition. Consistent with this view, the administration of IL-33, which has the ability to induce the production of Th2 cytokines, such as IL-3, IL-5, and IL-10, reduced the fat mass and adipose tissue hypertrophy in genetically obese ob/ob mice [10]. This was associated with a reduction of total cholesterol [10]. Overall, IL-33 effects have been attributed to the recruitment of beige adipocytes in the adipose tissue, a process known as “beiging” or “browning of fat” that regulates energy expenditure [170].

### 4.4. Anti-Inflammatory Effects of Triglyceride-Lowering Drugs

In the last decade, it has been shown that the beneficial cardiovascular outcomes of statin therapy were due not only to the reduction of cholesterol, but also to its anti-inflammatory actions independent of LDL levels [65]. There are three drug classes that are clinically available for hypertriglyceridemia treatment—fibrates, niacin, and omega-3 fatty acids [104]. Fibrates (gemfibrozil and fenofibrate) act via the peroxisome proliferator receptor selective for the alpha receptor (PPARα). They increase FFA oxidation, increase LPL synthesis, decrease apoCIII, increase apoAI and apoAII, with subsequent VLDL reduction. Overall, fibrates lower triglycerides by 30–50% and raise HDL by 10–20%. Niacin or nicotinic acid is a B-complex that inhibits the mobilization of FFA from peripheral tissues in basal and noradrenaline-stimulated conditions. It decreases triglyceride synthesis and increases apoB degradation, leading to a reduction of circulating VLDL. Moreover, niacin inhibits HDL catabolism, leading to a decrease of triglycerides by 10 to 30%, but also an increase of HDL-C by 10 to 40%. Polyunsaturated omega-3 fatty acids (PUFA), such as eicosapentaenoic acid (EPA) and docosahexaenoic acid (DHA), seem to promote ApoB degradation and facilitate chylomicron clearance, whereby they lower triglyceride levels. 

All these triglyceride-lowering drugs have shown to protect against CVD, even though the question as to whether hypertriglyceridemia is an independent risk factor for CVD remains unresolved [171]. With respect to the trials on the cardiovascular benefits of fibrates, in the Helsinki Heart Study [172] fibrates (gemfibrozil) significantly reduced cardiovascular events with no differences in cardiovascular and total mortality. In the Veterans Affairs High-Density Lipoprotein Cholesterol Intervention Trial (VA-HIT) [173], gemfibrozil significantly reduced nonfatal myocardial infarction, but did not change mortality rate. Interestingly, in that study, only HDL (and not triglycerides) predicted cardiovascular events. Then, the Bezafibrate Infarction Prevention Study showed that only in patients with triglycerides greater than 200 mg/dL, benzafibrate significantly reduced myocardial infarction and sudden death [174]. Last, the FIELD study demonstrated that fenofibrate significantly reduced non-fatal myocardial infarction and revascularization with no effects on total and cardiovascular mortality [175]. Consistent with these results, a recent meta-analysis has confirmed that fibrates can reduce the risk of major cardiovascular events, predominantly by prevention of coronary events [176]. With respect to the other lipid-lowering drugs, clinical trials using niacin, alone or in combination, with other lipid medications, have shown benefits in decreasing cardiovascular event rates and atherosclerosis [104]. Likewise, also PUFA exhibit cardioprotective effects, as their intake has shown to reduce the risk of cardiovascular disease in primary [177] and secondary prevention [178,179], as well as sudden death [180].

To date, it is not clear if the cardiovascular outcomes of triglyceride-lowering drugs are due to the reduction of lipid, inflammation, or both. Certainly, be it fibrates, niacin, or PUFA, all these drugs exhibit anti-inflammatory properties. For instance, fenofibrate can inhibit IL-6 expression in vascular smooth muscle cells and aortic explants [181,182], via a suppression of the NF-κB and activator protein 1 (AP-1) transcription factors. Moreover, it could inhibit VCAM-1 and MCP-1 expression in endothelial cells [183,184]. Furthermore, niacin displayed anti-inflammatory effects, as it significantly reduced the expression of fractalkine, MCP-1, RANTES, and iNOS in TNF-α-treated adipocytes, and it reduced macrophage migration [185]. As for PUFA, they exhibit anti-inflammatory properties, whereby they improve insulin sensitivity [186]. For instance, they are capable of inhibiting many aspects of leukocyte trafficking [187] and they inhibit TRL4 activation upon G-protein coupled receptor 120 (GPR120) binding [186]; they prevent activation of NLRP3 inflammasome in human monocytes/macrophages [188]; moreover, they can produce anti-inflammatory and inflammation resolving mediators called resolvins, protectins, and maresins [187]. These findings have shed light on the complexity of the interaction between nutrients and inflammation, but at the same time, they have open therapeutic perspectives against metabolic inflammation [37].

### 4.5. Lipids: Friends or Foes?

The VA-HIT study showed that raising HDL in patients with CVD significantly reduces the incidence of major coronary events [173]. The mechanisms underlying the benefits of raising HDL include not only the cholesterol efflux from the periphery to the liver, but also specific anti-inflammatory effects of HDL. It has been argued that HDL might have actually belonged to the innate immune system [189]. For instance, HDL is able to bind LPS and inhibit LPS-induced inflammatory responses [190]. Likewise, HDL can bind to other bacterial products and limit their toxic effects [191]. Interestingly, recent studies have shown that preincubation of macrophages with HDL significantly increased the expression of Activating Transcription Factor 3 (ATF3), which is a negative transcriptor regulator, whereby HDL might prevent the TLR4-mediated activation of macrophage [192]. Another mechanism underlying HDL effects is its ability to significantly reduce membrane cholesterol-rich lipid rafts, which are essential for macrophage response to TLR ligands [193,194]. Consistent with the modulation of macrophage activation, it has been reported that HDL induced atherosclerosis regression and altered the inflammatory properties of plaque monocyte-derived cells in *apoE*-knockout mice [195]. Likewise, apoAI, the major apolipoprotein of HDL, prevented T-cell activation and proliferation [196].

Recent studies have revealed that also other lipids—called lipokines—matter in the resolution of inflammation. The paradigm of these protective mediators is represented by the omega-3 fatty acids, which interact with the lipid sensor GPR120 with subsequent inhibition of TNF-α and TLR4-mediated inflammation [186]. The observation that a dysfunctional variant of GPR120 is associated with obesity, highlights the importance of this pathway and the possibility to use GPR120 as a target against metabolic disturbances [197]. Additional lipokines include the fatty acid palmitoleate [198], which has the ability to ameliorate atherosclerosis [199], to reverse high-fat diet-induced proinflammatory macrophage shift [200], and to prevent skeletal muscle insulin resistance [201]. Another bioactive lipid signal that protects against adipose tissue inflammation and exhibit anti-diabetic properties is represented by the palmitic acid hydroxyl stearic acids [202,203]. Last, also the cannabinoid receptor type 2, which binds to monoacylglycerols, triggers an anti-inflammatory signaling cascade. 

## 5. Conclusions

Lipid metabolism and the immune system are intertwined. In the era of the obesity epidemic, lipids and interleukins represent key mediators of cardio-metabolic diseases. Basic and clinical studies continue to remind us of the importance and the complexity of the crosstalk between lipids and interleukins. Nevertheless, knowledge gaps remain and only a deeper understanding of the crosstalk between these two systems might allow one to find better targeted anti-inflammatory therapies against CVD or diabetes. Further studies might discover cytokines with positive metabolic effects, as well as new lipid mediators with anti-inflammatory effects, which could represent new promising therapeutic tools.
